# Supplementary material for: Predicting the efficiency of oxygen-evolving electrolysis on the Moon and Mars
Source: Nat Commun. 2022 Feb 8;13:583. doi: 10.1038/s41467-022-28147-5 (PMC8826910; doi:10.1038/s41467-022-28147-5)
Supplement: Supplementary file 1 — Supplementary Information [file 41467_2022_28147_MOESM1_ESM.pdf]

Supplementary Information for:

Predicting the efficiency of oxygen-evolving electrolysis on the Moon and Mars

Bethany A. Lomax[1,2*], Gunter H. Just[3], Patrick J. McHugh[1], Paul K. Broadley[3], Greg C. Hutchings[3], Paul A. Burke[4], Matthew J. Roy[3], Katharine L. Smith[3], Mark D. Symes[1*]

[1] *WestCHEM, School of Chemistry, University of Glasgow, Glasgow, G12 8QQ, United Kingdom*

[2] *European Space Research and Technology Centre, Keplerlaan, Noordwijk, 2201 AZ, Netherlands*

[3] *Department of Mechanical, Aerospace and Civil Engineering, University of Manchester, Oxford Road, Manchester, M13 9PL, United Kingdom*

[4] *Space Exploration Sector, Johns Hopkins University Applied Physics Laboratory, Laurel, Maryland, United States of America*

**Email: beth.lomax@esa.int; mark.symes@glasgow.ac.uk*

Index	***Page***
Supplementary Figures	S3
Supplementary Table	S16
Supplementary Notes	S17
Supplementary References	S23

Supplementary Figures

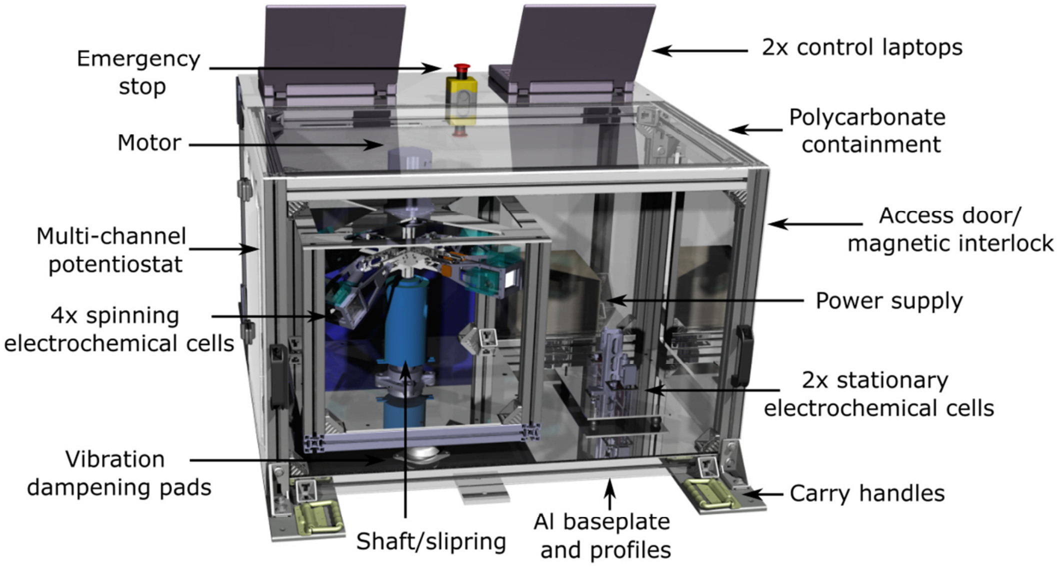

Supplementary Figure 1: CAD rendering of the rack designed for investigating altered-gravity electrolysis during parabolic flight. *The locations of the centrifuge, stationary cell table, and control equipment are marked.*

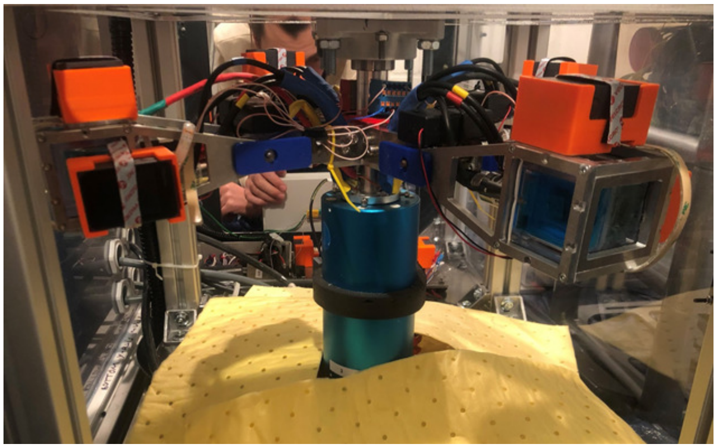

Supplementary Figure 2: The centrifuge used to create artificial gravity in flight configuration. *The photograph shows the centrifuge in a locked horizontal position showing the cell baskets, the camera holders, the cabling system, and the slip-ring.*

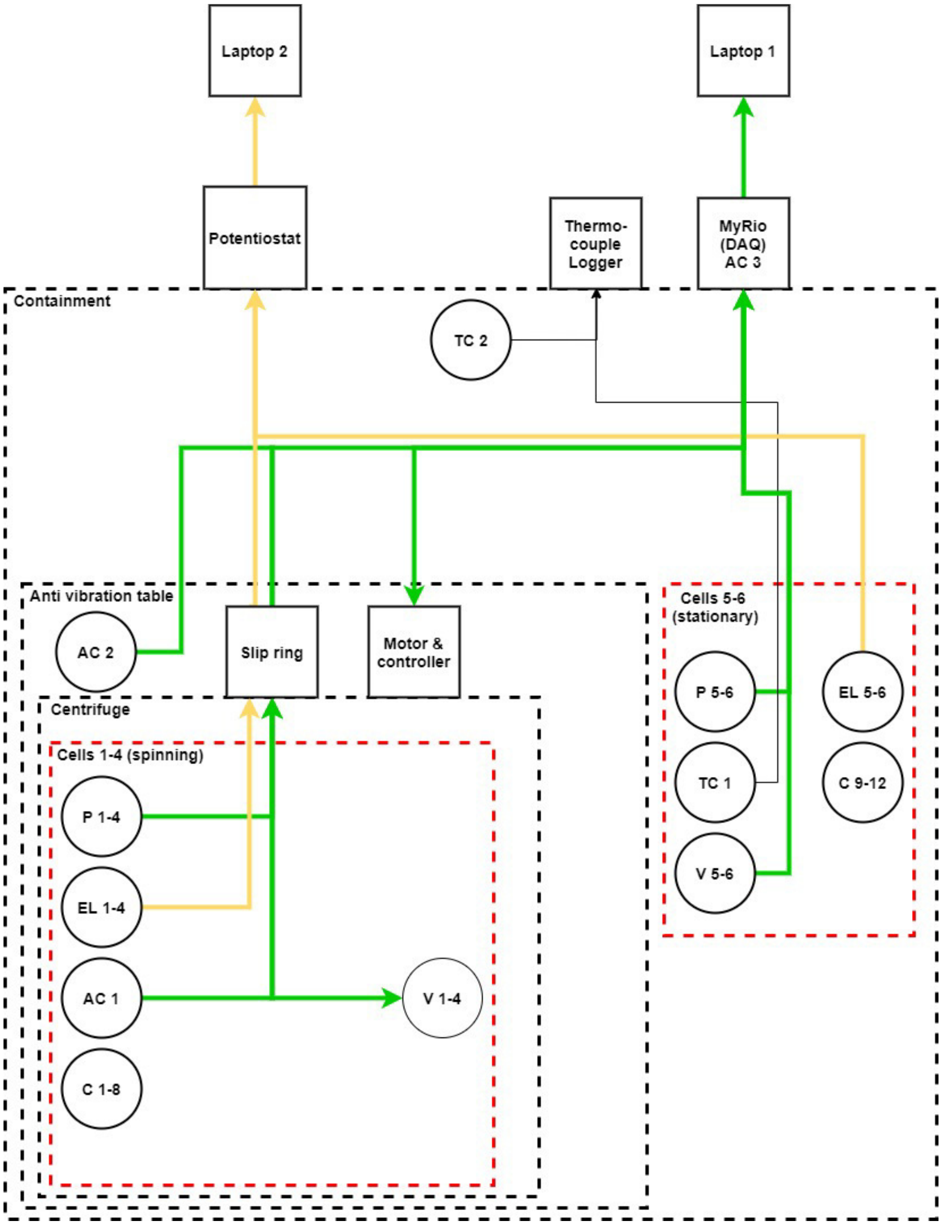

Supplementary Figure 3: Schematic of the DAQ and control system. *The schematic also includes an indication of system boundaries. Green arrows indicate a connection to Laptop 1 (rack control and DAQ), orange arrows indicate connection to Laptop 2 (electrolysis control and DAQ). Input controls and data on each laptop were handled independently. AC: accelerometer; P: pressure sensor; V: release valve; EL: electrochemical cell; TC: thermocouple; C: camera.*

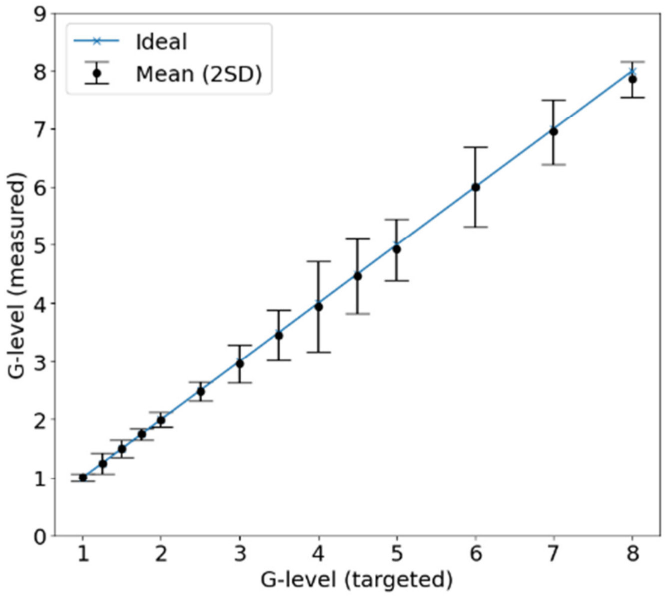

Supplementary Figure 4: Targeted vs. measured G-levels at 50 mA cm^{-2}. *The measured mean artificial acceleration acting on the cell for each of the targeted g-levels on the centrifuge during data collection at 50 mA cm^{-2}. Error bars represent ±2 standard deviations from the mean. Source data are provided as a Source Data file.*

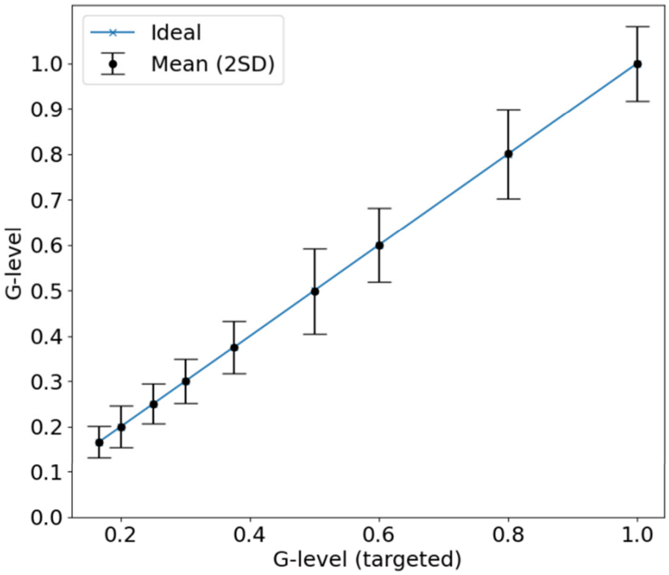

Supplementary Figure 5: Targeted vs. measured G-levels at 100 mA cm^{-2}. *The measured mean artificial acceleration acting on the cell for each of the targeted reduced gravity levels on the centrifuge for a representative data set collected at 100 mA cm^{-2}; error bars represent ±2 standard deviations from the mean. Source data are provided as a Source Data file.*

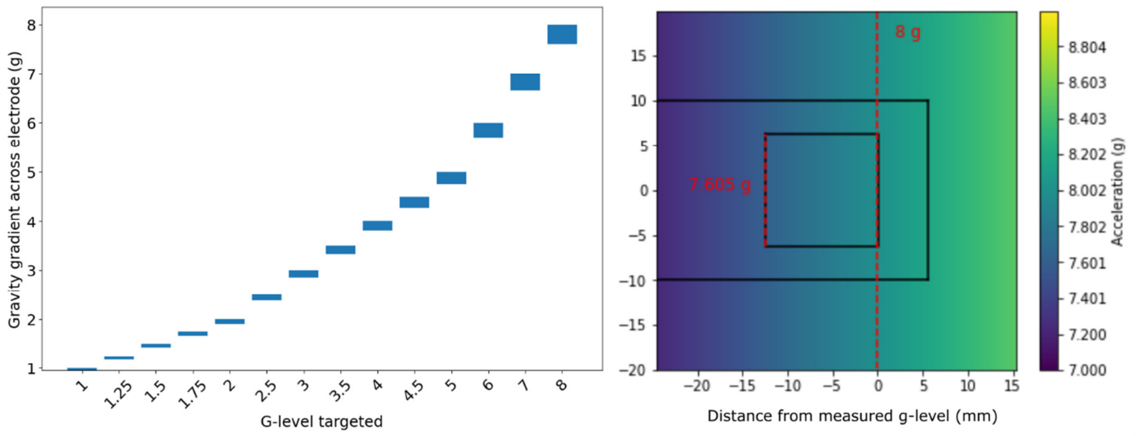

Supplementary Figure 6: Gravity gradients across the electrode for 1-8 g. *The calculated gravity gradient across the gas-evolving electrode surface for each of the targeted g-levels from 1-8 g (left) and an example (8 g) of the gravity gradient calculation across an intersection of the electrolysis cell along the face of the electrode where the variation in gravity is depicted by a color bar representing ±1 g (right). Source data are provided as a Source Data file.*

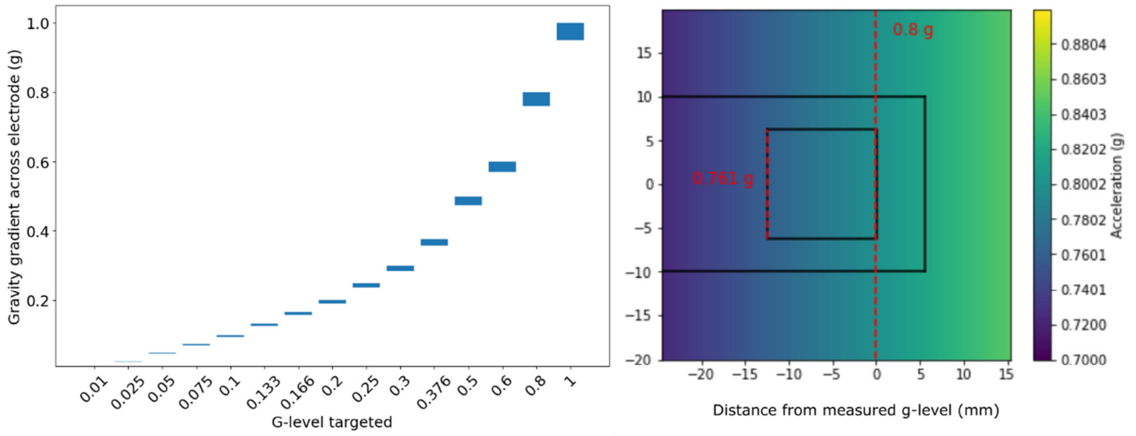

Supplementary Figure 7: Gravity gradients across the electrode for 0-1 g. *The calculated gravity gradient across the gas-evolving electrode surface for each of the targeted g-levels from 0-1 g (left) and an example (0.8 g) of the gravity gradient calculation across an intersection of the electrolysis cell along the face of the electrode where the variation in gravity is depicted by a color bar representing ±0.1 g (right). Source data are provided as a Source Data file.*

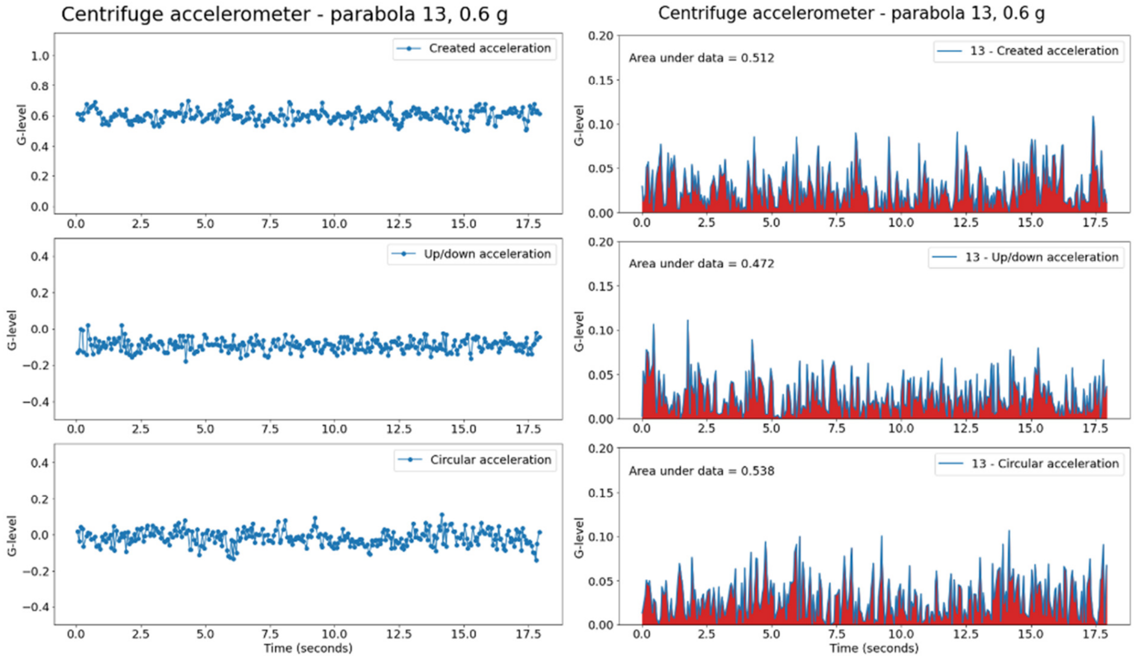

Supplementary Figure 8: Example data for vibrational analysis. *The raw accelerometer data (left) and processed vibrational intensity analysis (right) for an example experiment (0.6 g, 50 mA cm^{-1}) from the centrifuge operating on the parabolic flight. The total area shown in red is used to represent the vibrational intensity. Source data are provided as a Source Data file.*

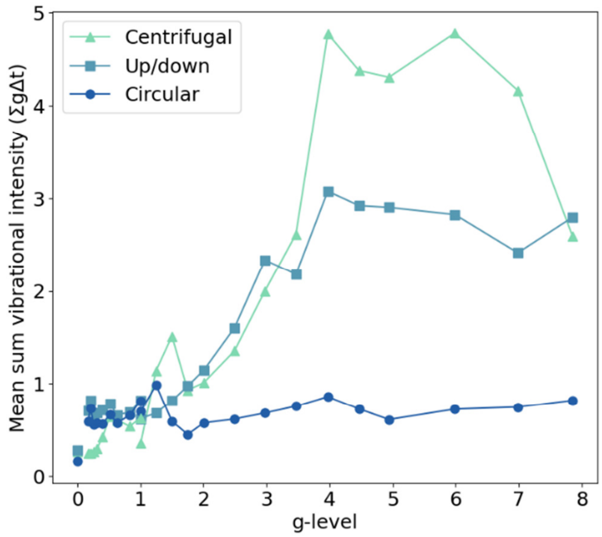

Supplementary Figure 9: comparison of the overall motion experienced by a cell across different experiments. *The average sum vibrational intensity experienced by the cells in three axes during hypergravity experiments on the short-arm centrifuge. Assuming the arm is extended horizontally, the centrifugal axis is that of the created acceleration: up/down is parallel to Earth's gravity, and circular is in the plane of rotation. Source data are provided as a Source Data file.*

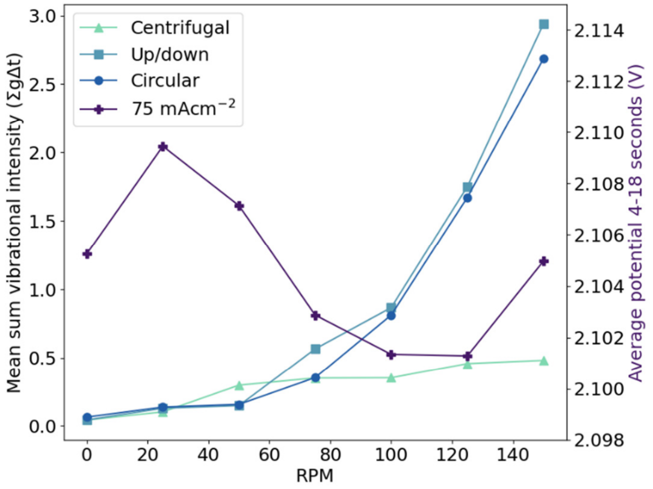

Supplementary Figure 10: The average sum vibrational intensity experienced by the cells in three axes during orbital shaker plate experiments under galvanostatic control. *The vibrational data (centrifugal, up/down, and circular) is represented by the left axis, and the electrochemical data is represented by the right axis. Source data are provided as a Source Data file.*

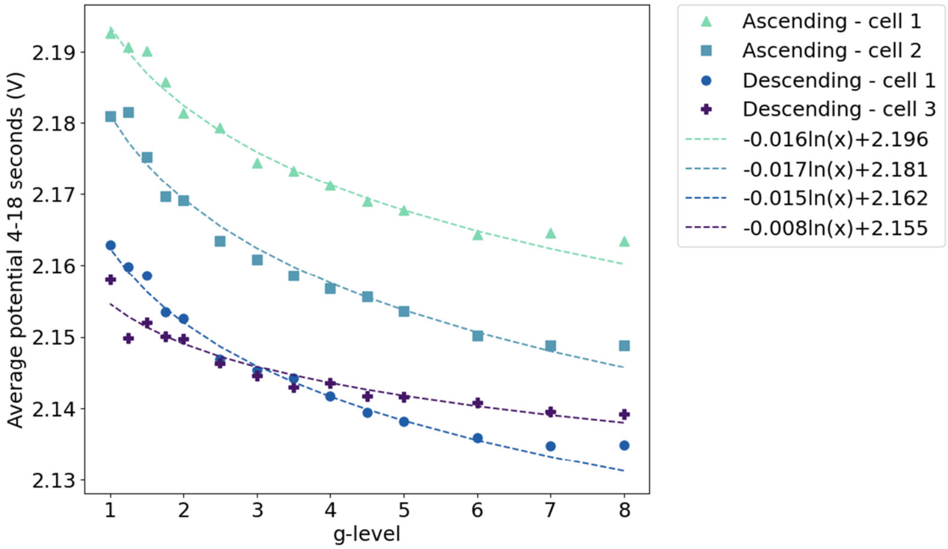

Supplementary Figure 11: Average potential vs g-level for 1-8 g. *The average potential at 100 mA cm^{-2}collected across 1 - 8 g with the short arm centrifuge where g-level order was either ascending (●) or descending (▪). R^2 values were >0.975 and >0.924 for the ascending and descending data fits respectively. Source data are provided as a Source Data file.*

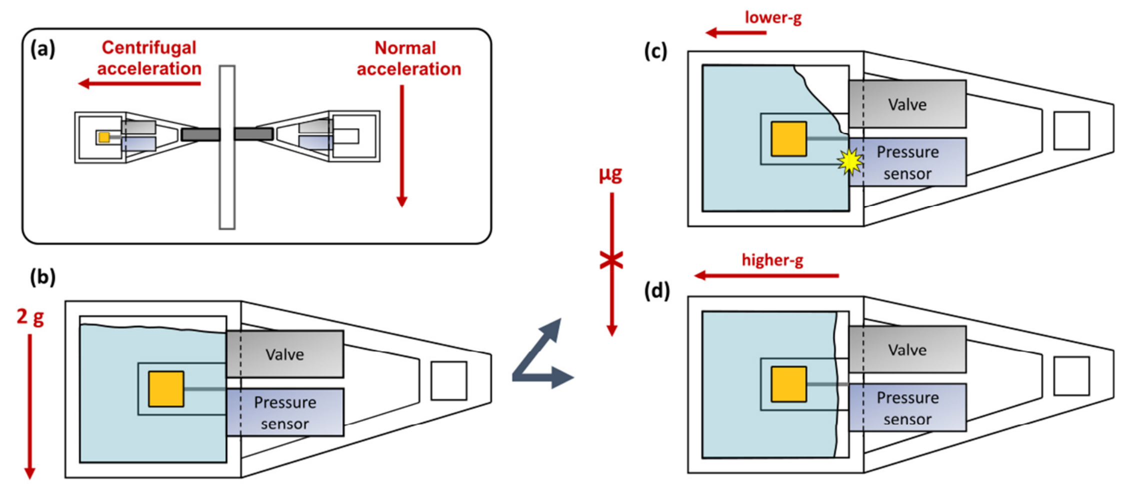

Supplementary Figure 12: Explanation of current loss via the pressure sensor ground connection at low g. *The figure shows the orientation of the baskets on the centrifuge (a), the cell and baskets in the 2-g phase prior to each parabola (b), and the transition to microgravity where a low (c) or high (d) g-level is artificially created. At the lower g-levels, the centrifugal acceleration is not strong enough to overcome the surface tension between the wall and electrolyte and completely re-orientate the headspace, causing the electrolyte to contact the sensor during electrolysis.*

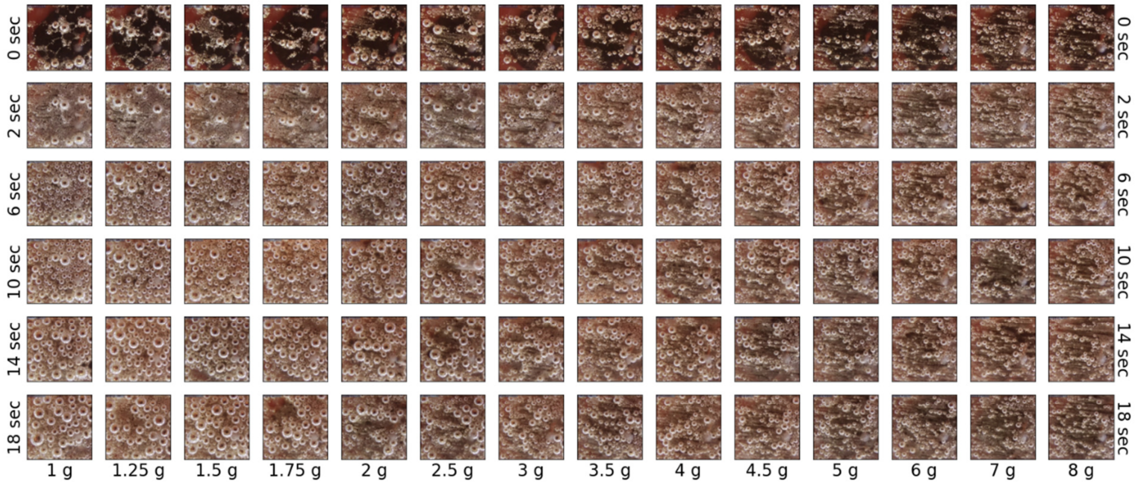

Supplementary Figure 13: Stills from video footage of the electrode at various times for 1-8 g. *A comparison of 1 cm^2 frames of the bubbles on the electrode surface across 1 - 8 g for a 50 mA cm^{-2} data set obtained on the centrifuge.*

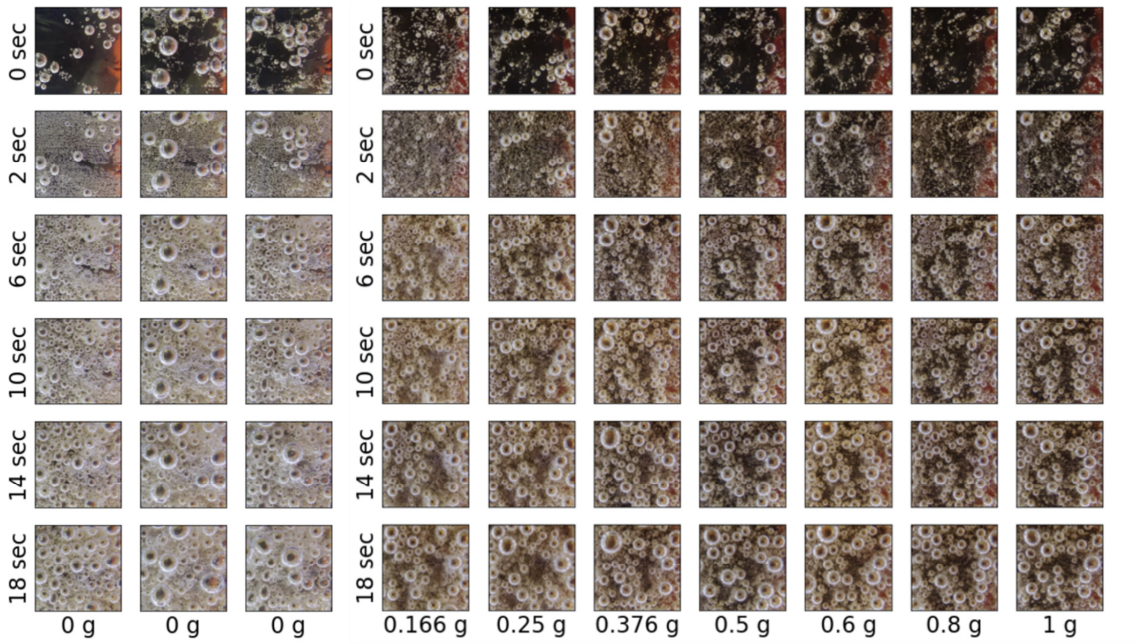

Supplementary Figure 14: Stills from video footage of the electrode at various times for 0-1 g. *A comparison of the bubble formation during electrolysis (t = 0-18 seconds) at 50 mA cm^{-2} under reduced gravity conditions. Three examples of different experiments in microgravity (left) show that bubble retention on the electrode surface increases as buoyancy forces become less important.*

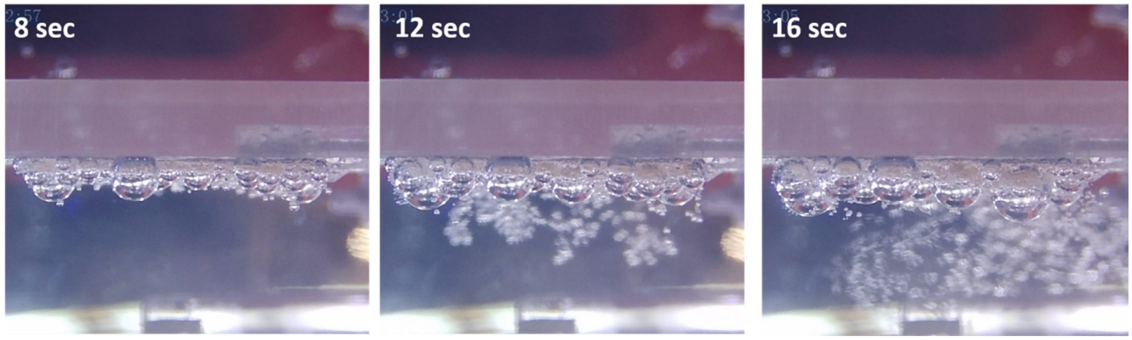

Supplementary Figure 15: Effect of g-jitter on small bubbles. *Small bubbles impacted by g-jitter on an electrode at 8, 12, and 16 seconds during an electrolysis carried out at 100 mA cm^{-2} in microgravity during parabolic flight.*

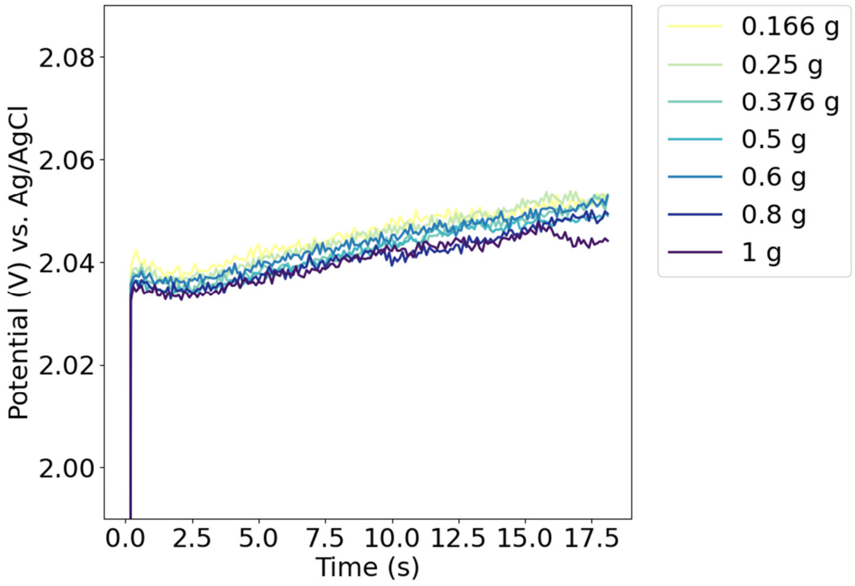

Supplementary Figure 16: Example raw V-t data at 50 mA cm^{-2}. *An example reduced-gravity data set collected using chronopotentiometry at 50 mA cm^{-2}. Source data are provided as a Source Data file.*

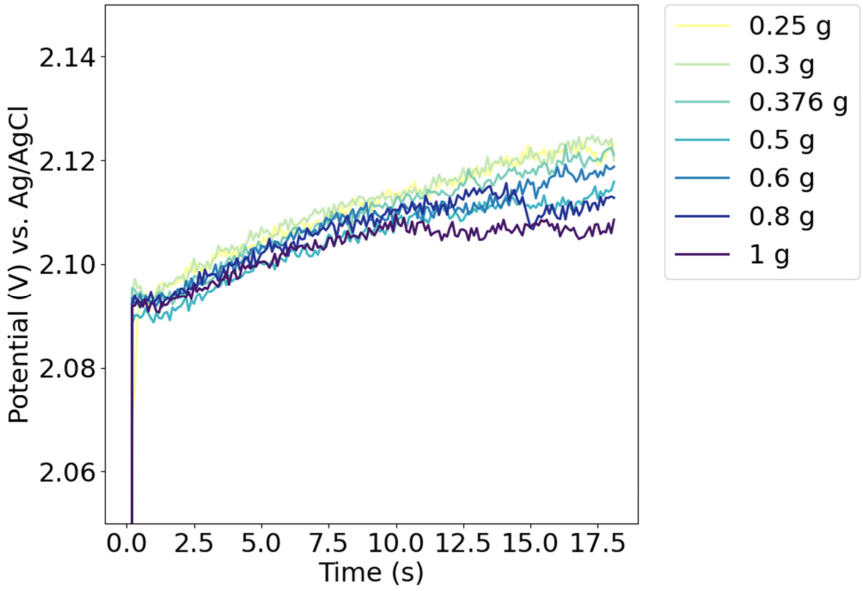

Supplementary Figure 17: Example raw V-t data at 75 mA cm^{-2}. *An example reduced-gravity data set collected using chronopotentiometry at 75 mA cm^{-2}. Source data are provided as a Source Data file.*

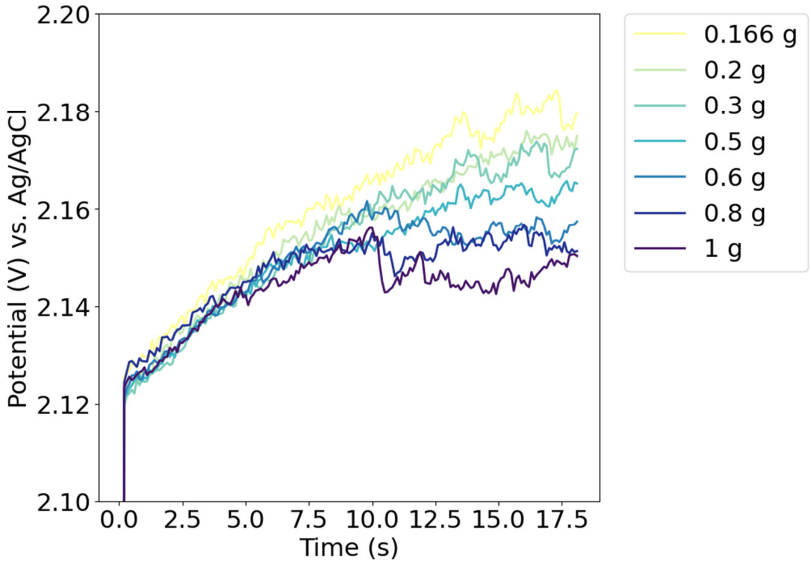

Supplementary Figure 18: Example raw V-t data at 100 mA cm^{-2}. *An example reduced-gravity data set collected using chronopotentiometry at 100 mAcm^{-2}. Source data are provided as a Source Data file.*

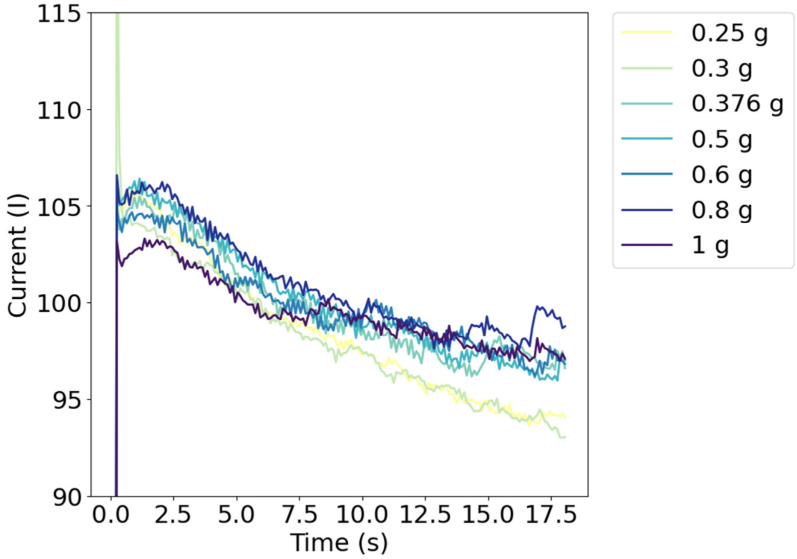

Supplementary Figure 19: Example raw i-t data at 2.075 V. *An example reduced-gravity data set collected using chronoamperometry at 2.075 V vs. Ag/AgCl. Source data are provided as a Source Data file.*

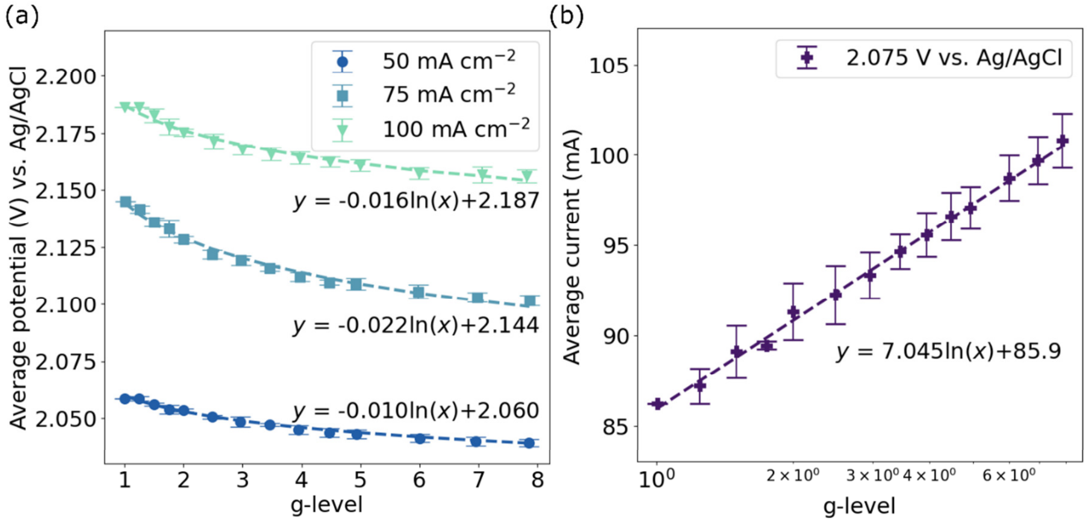

Supplementary Figure 20: Hypergravity gas-evolving electrolysis (1 – 8 g) data (Figure 2 in main text) displayed on a logarithmic scale. *The average potential during galvanostatic experiments (a) and average current during potentiostatic experiments (b) with hypergravity conditions achieved using a centrifuge. Error bars represent the standard error of the mean.* R^2 *values are >0.98 for all the fitted trends shown. Source data are provided as a Source Data file.*

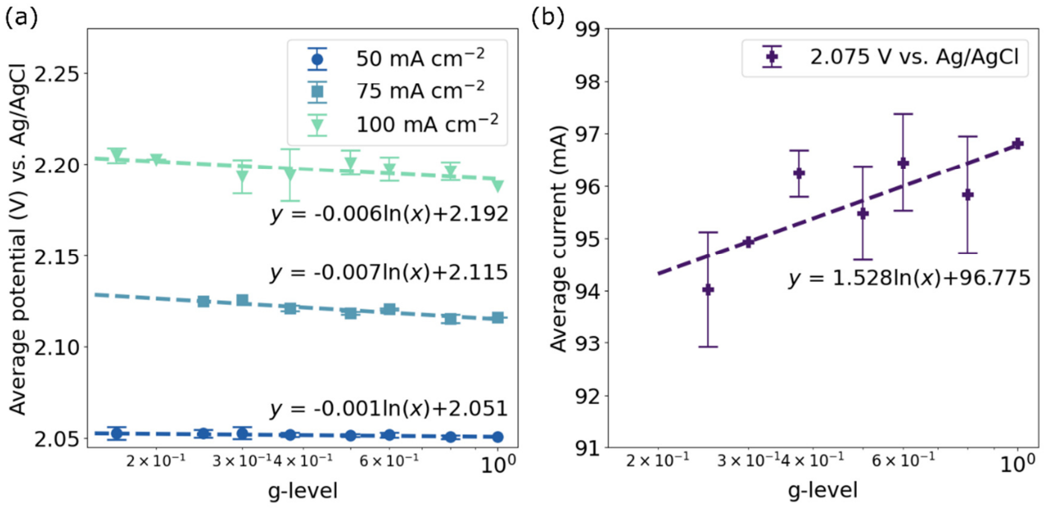

Supplementary Figure 21: Reduced gravity gas-evolving electrolysis (0 - 1 g) data (Figure 3 in main text) displayed on a logarithmic scale. *Figure 3 displayed on a logarithmic scale: The average potential or current of all reduced-gravity electrolysis experiments under galvanostatic (a) and potentiostatic (b) control, respectively, collected with the centrifuge. Error bars represent the standard error of the mean. Trends shown are calculated based on centrifuge data only and have an* R^2 *value of 0.848, 0.868, and 0.496 for 50, 75, and 100 mA cm*$^{-2}$ *respectively, and 0.651 for 2.075 V. Source data are provided as a Source Data file.*

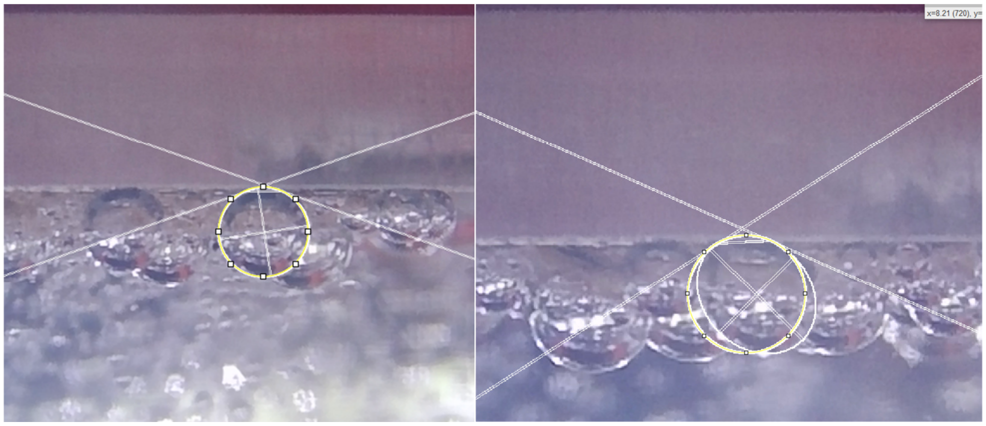

Supplementary Figure 22: Example contact angle data. *An example of the ImageJ contact angle measurement for a bubble at microgravity (left) and 1 g (right)*

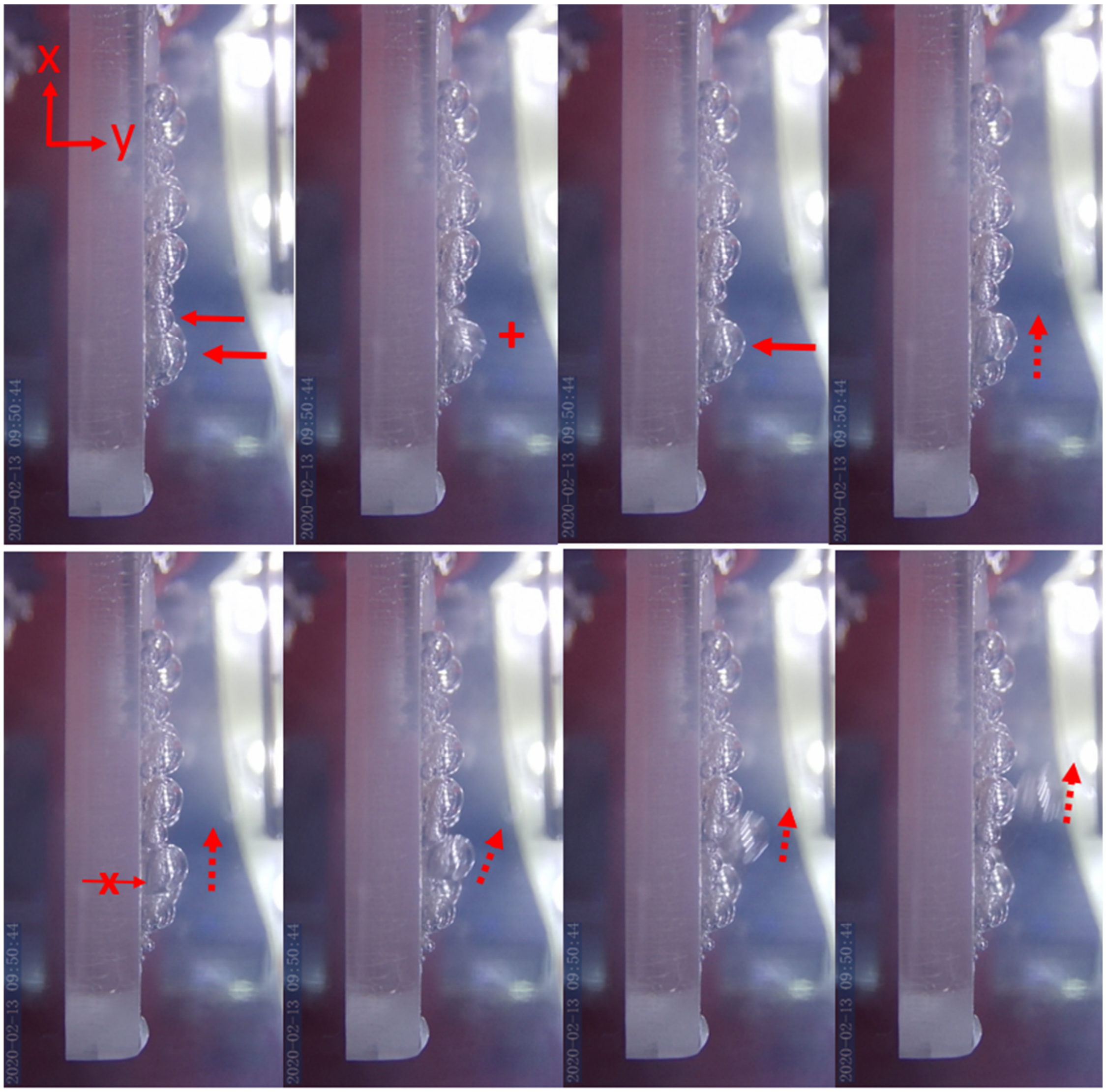

Supplementary Figure 23: Bubble behavior on the electrode surface. *One medium-large bubble and one smaller bubble coalesce, within <1 second the larger bubble that forms becomes unstable and slides along the face of the electrode. Here the force balance in the x direction is in the favour of buoyancy/departure. Then, once the advancing angle (the one at the back growing larger) is unsustainable, detachment occurs there initially, and the bubble then lifts off the surface in the y direction. Images are ordered sequentially left to right, where the second row follows directly from the end of the first row.*

Supplementary Table

Supplementary Table 1: Average advancing and receding contact angles for oxygen bubbles on the gold electrode surface. *Angles were measured in 1 g (n = 8) and micro-g (n = 8) conditions; angle labels correspond to those shown in Figure 6. The values shown in parentheses are the standard errors of the corresponding means.*

	1 g			0 g		
	θ_a (°)	θ_r (°)	$\theta_a - \theta_r$ (°)	θ_a (°)	θ_r (°)	$\theta_a - \theta_r$ (°)
6 seconds	39.81 (2.21)	38.11 (1.60)	1.70	28.02 (1.67)	28.04 (1.50)	-0.02
16 seconds	48.53 (6.07)	40.65 (5.31)	7.88	19.85 (0.92)	19.94 (0.98)	-0.09
Average	41.78			23.96		

Supplementary Notes

Supplementary Note 1

The first thing to consider when assessing the quality of data obtained on the short-arm centrifuge is the accuracy of the artificial g-levels created. On a short-arm centrifuge system, variation in radius can have a significant impact on the g-level; the calculated gravity gradient across the electrode is shown for all reduced- and hyper- gravity levels targeted on the centrifuge in Supplementary Figures 6 and 7, respectively. Assessment of the gravity gradient shows that no two-target g-levels overlap; a maximum Δg of 0.4 g when 8 g is created can be anticipated during the hypergravity experiments, and a maximum Δg of 0.049 g can be anticipated when 1 g is generated by the centrifuge during reduced-gravity experiments. The measured g-level is the maximum value across the electrode face. The measured acceleration experienced by the cell in the z-axis (parallel to the face of the electrode) is shown in Supplementary Figures 4 and 5 for hypergravity and reduced-gravity data respectively. Overall, the mean g-level achieved with the short-arm centrifuge was very close to the targeted values due to the successful control of the motor rotation rate via a closed PID feedback loop with the cell accelerometer. Error bars representing two standard deviations show that the spread of data around the mean generally increases with increasing g-level due to increasing vibration. While the error bars are partially overlapping between adjacent g-levels, they are never entirely concurrent, indicating that the experimental g-levels were distinct.

Supplementary Note 2

The vibrational intensity in all three axes for each g-level studied is quantified and compared in Supplementary Figure 9. The electrochemical cell on the short-arm centrifuge experienced a maximum vibrational intensity when rotating to create 4 g, with the faster rotational speeds appearing to stabilize the centrifuge arms slightly. Vibration levels in reduced-gravity experiments were significantly lower and more consistent than hypergravity experiments. The shaking motion of the cells may improve mass transport in the electrolyte and could influence the bubble detachment behavior. It has been shown that, while application of ultrasonic vibration can enhance hydrogen evolution, it in fact hinders oxygen evolution by limiting the coalescence and detachment of bubbles [1]. The frequency of the vibrations in the present study was approximately 5 - 8 Hz. This is evidently several orders of magnitude different to the ultrasonic vibration used in the aforementioned study, however, that work highlights that the relationship between the hydrodynamic behavior of bubbles and vibrations is complex and is highly dependent on the specifics of an electrochemical system such

as electrode surface properties, the gas and electrolyte composition, and their interaction at the three-phase boundaries.

To assess whether the low frequency vibration experienced by the electrochemical cells may have influenced the trends in electrochemical efficiency seen in the present study, data was collected at 75 mA cm^{-2} on an orbital shaking plate rotating between 0 and 150 RPM. The vibrational intensity was comparable to the centrifuge data and is shown for all three axes in Supplementary Figure 10. The calculated average peaks per second varied between 8 and 14 Hz across the data set, which is on the same order of magnitude as that seen on the short-arm centrifuge. Supplementary Figure 10 shows that the average potential of data collected with a fixed current density of 75 mA cm^{-2} varies by less than 10 mV and does not follow a clear trend with the increasing vibration. The total variation between 1 – 8 g in Figure 2 in the main text is between 20 and 45 mV. While any level of varying vibration may influence electrochemical data, the comparison with data on the orbital shaking plate confirms that it cannot entirely account for the electrolysis trends that were identified.

Supplementary Note 3

Additionally, it was important to exclude a change in electrolyte composition as the primary cause for the gravity-dependent trend. While variation is unavoidable as Cu is removed from the electrolyte at the cathode and H^+ is generated at the anode, the electrolyte concentrations were chosen such that the excess of these species would mean that the change across a set of 15 experiments would be as small as possible. The upper limitations were solubility and flight safety regulations for the concentrations of copper sulfate and sulfuric acid, respectively. Supplementary Figure 11 shows hypergravity data collected at 100 mA cm^{-2} where the g-level was varied in both ascending and descending order. The same trend is seen in all data sets, with two ascending and one descending set following an almost identical logarithmic fit. One descending data set has a slightly shallower trend, indicating that the potential may slightly decrease over time in the system, but not enough to account for the trends seen in the hypergravity data, which validates the use of the chosen electrolyte composition to limit concentration variation. This data was collected with the highest current density used in this work; therefore, the effect would theoretically be lesser at all other current densities. As discussed in the main text, the shifted baselines seen in Supplementary Figure 11 are most likely due to small differences between the electrodes in each individual cell; this shift can be accounted for when considering the percentage change relative to 1 g in an individual dataset.

Supplementary Note 4

Other forces specific to centrifuge systems should be considered when using a short-arm centrifuge, such as the contribution of shear forces as gravity changes laterally across flat surfaces [2]. As the gas-evolving electrode face of interest was not horizontal relative to the axis of rotation but rather perpendicular to the plane of rotation and parallel to the radius, the impact of lateral shear forces can be considered negligible. Additionally, as the centrifuge is a spinning system, the Coriolis force could potentially influence the bubble behavior following detachment from the electrode surface. As such, all experiments were conducted with the gas-evolving electrode facing away from the direction of rotation so detached bubbles were less likely to disturb the electrode surface if influenced by the Coriolis force.

Flight data may also be affected by the bubble attachment behavior observed in reduced-gravity. The contact angle of a bubble on a vertical surface under low-gravity conditions is smaller than at higher g due to lower hydrostatic pressure; additionally, less vertical buoyancy force results in less difference between the advancing and receding contact angles [3], [4], [5]. The interfacial tension force that contributes to keeping a bubble attached to a surface is a function of the contact angles and contact diameter [6]. It is feasible that the lower interfacial tension force in reduced-gravity conditions means that the bubbles are more susceptible to influence from external factors, such as vibration from the centrifuge or g-jitter from the aircraft, which could preferentially expedite detachment of smaller bubbles. Additionally, as the cells were fixed in a horizontal orientation, the transition from 2 g to microgravity at the start of each parabola resulted in the complete reorientation of the electrolyte just prior to electrolysis. This sloshing motion may have impacted the results by introducing flow in the electrolyte and improving mass transport in the cell. If the impact of external factors on detachment was greater at lower g-levels, that would serve to oppose the influence of reduced buoyancy force and could dampen the apparent loss of efficiency at lower g-levels. Further work in more stable reduced-gravity conditions, such as those offered by a drop-tower, could potentially reduce this source of error.

Supplementary Note 5

Video footage suggests that g-jitter during the microgravity parabolas helped to dislodge smaller bubbles from the surface prematurely. Supplementary Figure 15 shows sequential images of bubbles during an experiment in microgravity, where the small bubbles are dislodged from the surface while the larger bubbles are less impacted. As some of the bubbles, particularly the smaller ones, are not attached to the surface but rather floating in the vicinity of the electrode, they will generate less

ohmic resistance. Previous microgravity studies in a drop tower have found the ohmic resistance at a gas-evolving electrode to increase steadily over time as more bubbles accumulate at the surface and block the electrode [7], [8]. In the present work, no significant increase in resistance over the course of an experiment was seen in microgravity. While microgravity electrolysis was not the primary focus of the present study, this effect may also be present in the reduced-gravity data. Further, these results highlight why the majority of microgravity electrolysis work is carried out in drop towers rather than parabolic flights, as a much higher quality of microgravity is possible with drop towers [7], [8], [9], [10].

Supplementary Note 6

ImageJ Contact Angle plug-in (https://imagej.nih.gov/ij/plugins/contact-angle.html) was used to investigate the contact angle of bubbles in different gravity levels. First, ImageJ was calibrated with the known thickness of 3 mm for the electrode holder. Then, the two contact points of the bubble with the surface were selected, before 5 further points were selected along the bubble perimeter. The plug-in then calculates both advancing (left) as well as receding (right) contact angles by using the sphere approximation (ϑ=2$a$tan(2h/l)). The plug-in provides the internal angles, which would be relevant to a liquid drop, therefore, the supplementary angle was calculated to be relevant for the submerged bubble. Each bubble was measured three times and an average of these measurements was used. Then the mean and standard errors of the advancing and receding contact angles were calculated for 0 g and 1 g at 6 and 16 seconds, which is the data displayed in Table 1. An example of such analysis can be seen in Supplementary Figure 22.

Supplementary Note 7

Bubbles can be removed from the electrode in two ways. Bubble sliding, in which the bubble remains attached to the electrode, is the process by which a bubble slides vertically along the electrode until it meets the free surface. Secondly, bubbles can be removed from the electrode via the process of bubble detachment. During bubble detachment, bubbles, under the influence of buoyancy, neck and subsequently detach from the electrode and rise through the free liquid. These two processes can also both occur in succession, where a bubble first slides and subsequently lifts off the surface, as can be observed in Supplementary Figure 23. In microgravity, bubbles of roughly the same diameter following coalescence were stable on the surface and did not slide or lift off. When a bubble forms and grows on a surface which is not horizontal, it will grow asymmetrically and possibly slide along the inclined surface, while remaining attached [11]. The moment and bubble size

at which bubble sliding starts are dependent upon fluid properties (such as surface tension) and solid properties (such a surface energy and surface roughness). When bubbles slide vertically along the electrode's surface up to the free surface, they will coalesce with other bubbles, thus increasing bubble removal rate. Das et al. have shown that bubble sliding velocity and frequency of bubble sliding increases as the gravity vector increases [12]. This is caused by the increased buoyance force with increased gravitational acceleration. So, as gravity is decreased, bubble sliding frequency and velocity both decrease. Therefore, the bubble removal rate and electrolysis efficiency both decrease.

Supplementary Note 8

Past work has attempted to use various dimensionless quantities to scale bubble behavior across gravitational acceleration levels. However, the bubble behavior observed in the reduced gravity experiments cannot be fully accounted for with traditional scaling techniques. Pamperin, for example, used the Weber number to study bubble detachment from a submerged orifice in reduced gravity [13]. The Weber number is the ratio of inertial forces to cohesion forces (surface tension) acting on a multiphase flow (Eqn. 1) [13]. Although surface tension forces are expected to be significant in the reduced-gravity experiments described within this paper, the Weber number is not germane to the work described herein. The Weber number does not account for the change in gravitational acceleration. Secondly, bubbles nucleating, growing, and detaching through the process of electrolysis have minimal inertial forces acting upon them. Pamperin's experiments, by contrast, studied bubble detachment via gas jetting through an orifice [13].

$$We = \frac{\rho\, v^2\, l}{\sigma} \quad (1)$$

The Grashof number might also be a candidate dimensionless quantity to scale fluid flows across gravity levels. The Grashof number is a ratio of buoyant to viscous forces acting on a fluid flow (Eqn. 2) [14]. While it is relevant to buoyant flows, the Grashof number is typically used to study single-phase flows experiencing natural convection, caused by temperature gradients. In contrast, the experiment conducted for this research aimed to maintain constant temperatures. The buoyant flows, instead, were caused by the electrolytic nucleation of gas bubbles. Lastly, the research conducted herein did not study the influence of viscosity (the second main term in the Grashof number) on bubble nucleation and growth.

$$Gr = \frac{g\, \beta\, \Delta T\, l^3}{\vartheta^2} \quad (2)$$

The Froude Number is another dimensionless quantity frequently used to characterize the influence of gravity on a fluid flow. The Froude number is the ratio of inertial forces to external body forces, often simply defined as the body force due to gravity (Eqn. 3) [15]. It is interesting to note that the Froude number scales nonlinearly with gravity, similar to the nonlinear relationship found in the research discussed in this paper. However, similarly to the Weber number, the Froude number's main parameter focuses on a flow's inertial forces. Since electrolytic bubble nucleation and growth occur in a non-flowing liquid, the Froude number does not completely apply to our work.

$$Fr = \frac{u}{\sqrt{g\ l}} \qquad (3)$$

Finally, the Bond Number is perhaps the most frequently used dimensionless quantity when attempting to characterize bubble and droplet behavior, especially bubble shape. The Bond Number is the ratio of gravitational to surface tension forces (Eqn. 4) [16]. The Bond Number appears very applicable to the problem being studied: accounting for both the buoyant and surface-tension forces acting on the bubbles. However, there remain some important limitations to the use of the Bond Number. First, the Bond Number makes the assumption that the bubble is completely surrounded by liquid. That is, the Bond Number fails to account for any solid-fluid interactions. It has been shown that the properties of the solid, on which the bubble is adhered, can greatly influence the detachment time and volume of the bubble [10,17-20]. Since electrolysis efficiency is directly related to the release of bubbles from the electrode, the properties of the solid electrode, such as surface roughness or surface energy, must be accounted for in any dimensionless quantity used to scale across gravity levels. Finally, the Bond Number suggests a linear relationship as bubble behavior is scaled across gravity levels. The experimental trends presented in this research run counter to this, by displaying a nonlinear, logarithmic relationship between electrolysis efficiency and gravity level. Hence a dimensionless number that can accurately capture all the features required for modeling the behavior observed in this work, including the properties of the solid phase, has yet to be developed.

$$Bo = \frac{\Delta\rho\ g\ l^2}{\gamma} \qquad (4)$$

Supplementary References

[1] S. De Li, C. C. Wang, and C. Y. Chen, “Water electrolysis in the presence of an ultrasonic field,” *Electrochim. Acta*, vol. 54, no. 15, pp. 3877–3883, Jun. 2009, doi: 10.1016/j.electacta.2009.01.087.

[2] J. J. W. A. Van Loon, E. H. T. E. Folgering, C. V. C. Bouten, J. P. Veldhuijzen, and T. H. Smit, “Inertial shear forces and the use of centrifuges in gravity research. What is the proper control?,” *J. Biomech. Eng.*, vol. 125, no. 3, pp. 342–346, Jun. 2003, doi: 10.1115/1.1574521.

[3] A. Taqieddin, R. Nazari, L. Rajic, and A. Alshawabkeh, “Review—Physicochemical Hydrodynamics of Gas Bubbles in Two Phase Electrochemical Systems,” *J. Electrochem. Soc.*, vol. 164, no. 13, pp. E448–E459, 2017, doi: 10.1149/2.1161713jes.

[4] A. I. Rusanov, N. E. Esipova, and V. D. Sobolev, “Strong Dependence of Contact Angle on Pressure,” *Dokl. Phys. Chem.*, vol. 487, no. 1, pp. 87–90, Jul. 2019, doi: 10.1134/S0012501619070017.

[5] N. E. Esipova, A. I. Rusanov, V. D. Sobolev, and S. V. Itskov, “The Influence of Hydrostatic Pressure on the Contact Angle of a Sessile Bubble,” *Colloid J.*, vol. 81, no. 5, pp. 507–514, Sep. 2019, doi: 10.1134/S1061933X1905003X.

[6] D. Zhang and K. Zeng, “Evaluating the behavior of electrolytic gas bubbles and their effect on the cell voltage in alkaline water electrolysis,” *Ind. Eng. Chem. Res.*, vol. 51, no. 42, pp. 13825–13832, Oct. 2012, doi: 10.1021/ie301029e.

[7] D. Kiuchi, H. Matsushima, Y. Fukunaka, and K. Kuribayashi, “Ohmic Resistance Measurement of Bubble Froth Layer in Water Electrolysis under Microgravity,” *J. Electrochem. Soc.*, vol. 153, no. 8, p. E138, 2006, doi: 10.1149/1.2207008.

[8] G. Sakuma, Y. Fukunaka, and H. Matsushima, “Nucleation and growth of electrolytic gas bubbles under microgravity,” *Int. J. Hydrogen Energy*, vol. 39, no. 15, pp. 7638–7645, 2014, doi: 10.1016/j.ijhydene.2014.03.059.

[9] H. Matsushima, T. Nishida, Y. Konishi, Y. Fukunaka, Y. Ito, and K. Kuribayashi, “Water electrolysis under microgravity: Part 1. Experimental technique,” *Electrochim. Acta*, vol. 48, no. 28, pp. 4119–4125, 2003, doi: 10.1016/S0013-4686(03)00579-6.

[10] K. Brinkert *et al.*, “Efficient solar hydrogen generation in microgravity environment,” *Nat. Commun.*, vol. 9, no. 1, 2018, doi: 10.1038/s41467-018-04844-y.

[11] Das, A.K., and Das, P.K., "Bubble evolution and necking at a submerged orifice for the complete range of orifice tilt," AIChE Journal, Vol. 59, No. 2, 2013, pp. 630-642.

[12] Das, S., Weerasiri, L.D., and Yang, W., "Influence of surface tension on bubble nucleation, formation and onset of sliding," Colloids and Surfaces A: Physicochemical and Engineering Aspects, Vol. 516, 2017, pp. 23-31.

[13] Pamperin, O., and Rath, H., "Influence of buoyancy on bubble formation at submerged orifices," Chemical Engineering Science, Vol. 50, No. 19, 1995, pp. 3009-3024.

[14] Bergman, T.L., Lavine, A.S., Incropera, F.P., "Fundamentals of Heat and Mass Transfer," Wiley, Somerset, 2011, pp. 408.

[15] White, F.M., and Xue, H., "Fluid mechanics," McGraw-Hill, New York, NY, 2021, pp. 294.

[16] Clift, R., Grace, J.R., and Weber, M.E., "Bubbles, drops, and particles," Acad. Pr, New York, NY [u.a.], 1978, pp. 26.

[17] Burke, P.A., and Dunbar, B.J., "Development of Computational Fluid Dynamic (CFD) Models of the Formation and Buoyancy-Driven Detachment of Bubbles in Variable Gravity Environments," *AIAA Scitech 2021 Forum* (2021). DOI: 10.2514/6.2021-1838.

[18] Sakuma, G., Fukunaka, Y. & Matsushima, H. "Nucleation and growth of electrolytic gas bubbles under microgravity". Int. J. Hydrogen Energy 39, 7638–7645 (2014).

[19] Xu, W., Lu, Z., Sun, X., Jiang, L. & Duan, X. "Superwetting Electrodes for Gas-Involving Electrocatalysis". Acc. Chem. Res. 51, 1590–1598 (2018).

[20] Zhao, X., Ren, H. & Luo, L. "Gas Bubbles in Electrochemical Gas Evolution Reactions." Langmuir 35, 5392–5408 (2019).
